# The Sex Dependent and Independent Effects of Dietary Whey Proteins Are Passed from the Mother to the Offspring

**DOI:** 10.1002/mnfr.202400584

**Published:** 2024-11-03

**Authors:** Kanishka N. Nilaweera, Oleksandr Nychyk, William McCarthy, Luiza P. D. Moreira, Qusai M. Alabedallat, Deirdre Purfied, Jennifer Doyle, Paul Cormican, Antonia Santos, Xiaofei Yin, John Tobin, John R. Speakman, Donagh Berry, Lorraine Brennan, Paul D. Cotter

**Affiliations:** ^1^ Food Bioscience Department Teagasc Food Research Centre, Moorepark County Cork Fermoy P61 C996 Ireland; ^2^ VistaMilk Research Centre Teagasc, Moorepark County Cork Fermoy P61 C996 Ireland; ^3^ Food Chemistry and Technology Department Teagasc Food Research Centre, Moorepark County Cork Fermoy P61 C996 Ireland; ^4^ Animal Bioscience Department Teagasc Food Research Centre, Moorepark County Cork Fermoy P61 C996 Ireland; ^5^ Animal and Grassland Research and Innovation Centre Teagasc, Grange County Meath Dunsany C15 PW93 Ireland; ^6^ Teagasc Food Research Centre, Ashtown Dublin D15 DY05 Ireland; ^7^ School of Agriculture and Food Science Institute of Food and Health and Conway Institute, University College Dublin Dublin D04 C1P1 Ireland; ^8^ School of Biological Sciences University of Aberdeen Aberdeen AB24 2TZ UK; ^9^ APC Microbiome Ireland University College Cork Cork T12 YT20 Ireland

**Keywords:** Keywords, gut metabolome, macronutrient catabolism, reproduction, sex, transcriptome

## Abstract

**Scope:**

The study assesses the metabolic impact of dietary whey proteins across generations.

**Method and results:**

Virgin females are fed 20% energy whey proteins with 70% energy carbohydrates, which reduces body weight gain and visceral adipose compared to controls fed dietary casein. In contrast, the males are unresponsive. The effect is accentuated in reproductive females that also have reduced plasma levels of glucose. The responsive females have increased cecal levels of pyruvic and lactic acid, suggesting a greater catabolism of carbohydrates in the gut. While the male and female offspring born to mothers on whey proteins continue to reduce body weight gain, the female offspring further decreases the visceral and subcutaneous tissues and increases the gut capacity to breakdown dietary carbohydrates and proteins, whereas the male offspring are able to only decrease the visceral and increase protein catabolism in the gut. The ileum of male mice responded by reducing the gene expression for fibroblast growth factor 15 and increasing the expression of chymotrypsinogen B1.

**Conclusion:**

The effect of whey proteins on growth can be passed from the mother to the offspring without a sex preference, whereas the transmission of gut activity and adiposity are dependent on the sex of the offspring.

## Graphical abstract

In response to feeding dietary whey protein isolate (WPI), the impact on body weight in the parental generation is passed onto the offspring without a sex preference (panels 1–3). In contrast, the sex of the offspring influences the fat storage in the body and the gut capacity to breakdown carbohydrates (CHO) and proteins.

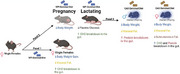

## 1 Introduction

The intake of energy dense foods has increased over the last century. This coupled, with the accompanying changes in the composition and functional potential of the gut microbiota, has led to metabolic disturbances in humans with effects on health.^[^ ^1^, ^2^, ^3^ ^]^ Altering the quality of the macronutrients in the diet can reduce or reverse some of these effects.^[^ ^4^, ^5^, ^6^ ^]^ Of note, we and others have shown the capacity of milk associated whey proteins to reduce body weight gain and adiposity and improve the metabolic health in humans and rodents consuming a high fat or high carbohydrate‐enriched diets.^[^ ^4^, ^5^, ^7^, ^8^ ^]^ The efficacy of whey proteins to achieve this effect can be related to the associated amino acid composition and quantity, how the protein is digested in the gut and absorbed compared to other dietary proteins including dietary casein.^[^ ^5^, ^7^, ^9^ ^]^

In searching for the mechanism for the protein‐effects, we have shown that dietary whey proteins (relative to casein) alter the composition of the gut microbiota and the nutrient and metabolites produced in the caecum of mice fed a high fat diet.^[^ ^5^, ^10^, ^11^, ^12^ ^]^ Introduction of antibiotics in drinking water depleted the gut microbiota and altered the cecal metabolome in mice, which were accompanied by a loss of specificity of dietary whey proteins to affect adiposity and the functionality of the gut.^[^ ^5^, ^10^ ^]^ Fecal transfer experiments showed the efficacy of the gut microbiota and associated metabolites to mediate the effects of dietary whey proteins on parameters related to growth.^[^ ^5^ ^]^ These findings, suggestive of a mechanism, were extended to show efficacy of whey proteins in diets enriched with carbohydrates.^[^ ^8^ ^]^

Based on the impact on the adipose tissues, we were the first to highlight that an interaction between whey proteins and the quantity of carbohydrates and fats in the diet occurs within the gut, and relative to casein, this interaction allows energy deposition and use in specific adipose tissues to be controlled by the diet and thereby the size (weight) of the tissues.^[^ ^7^ ^]^ The affected tissues were the subcutaneous white adipose tissue (sWAT) and the visceral white adipose tissues (vWAT), which appear to respond to the quality of the proteins and its interaction with dietary carbohydrates and fats (C/F ratio) in the diet.^[^ ^7^ ^]^ We provided further evidence that the effect on the adipose tissues is likely to be mediated by the gut microbiota and the metabolites produced by these micro‐organisms sensitive to dietary whey proteins.^[^ ^5^, ^7^ ^]^ Our discovery was significantly because it showed for the first time that the diet can be tailored to selectively deplete one adipose tissue without affecting the other, which can potentially impact on metabolic health, particularly if the vWAT can be selectively depleted.^[^ ^13^, ^14^ ^]^ Beyond the health impact, there is also a societal burden on males and females to have specific body shapes^[^ ^15^ ^]^ that we know to store excess energy in the vWAT (mainly males) and sWAT (mainly females).^[^ ^16^, ^17^, ^18^, ^19^ ^]^ To our knowledge, there are no dietary interventions that can selectively deplete the vWAT or sWAT.

**Figure 1:**
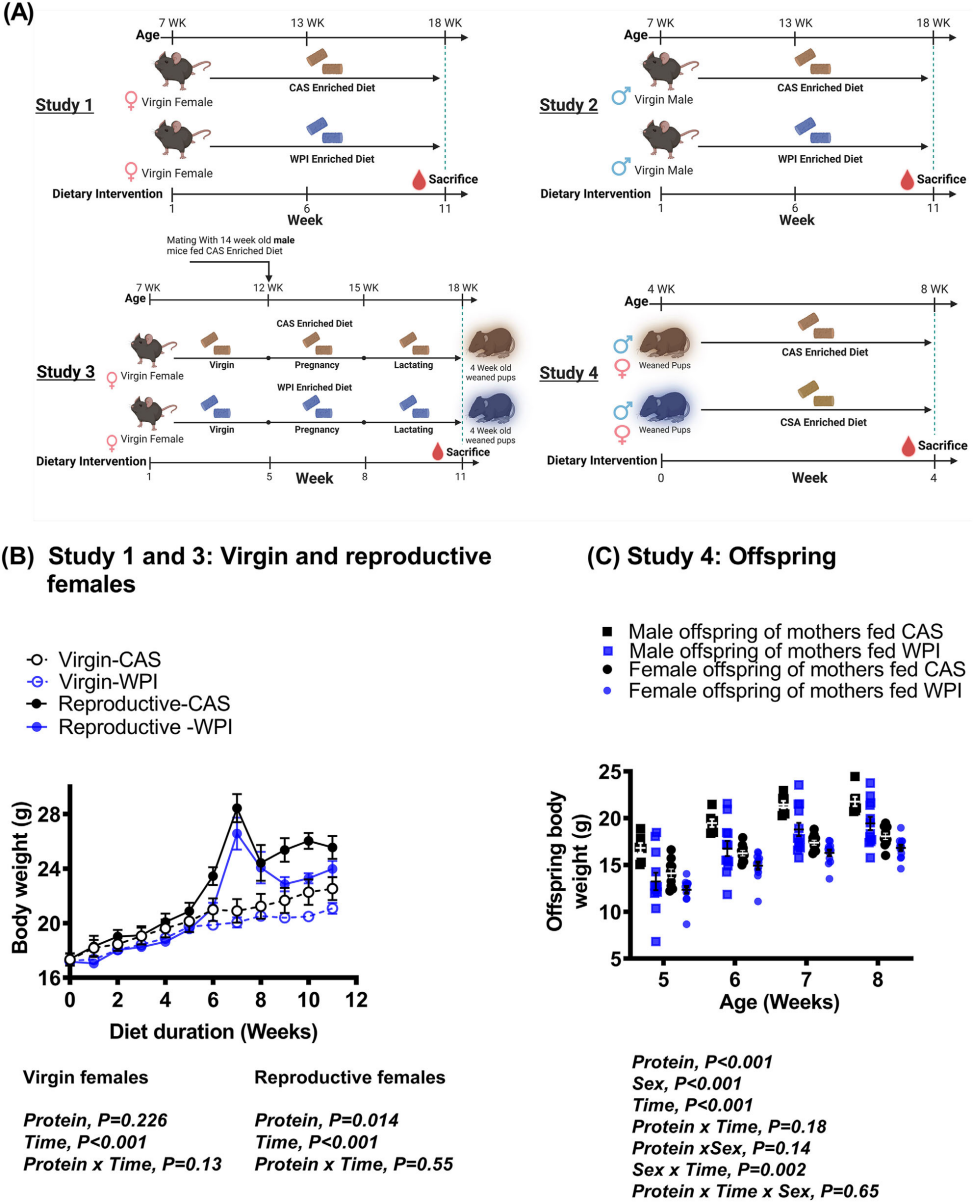
The impact of whey protein isolate (WPI) relative to casein (CAS) on body weight. The study design is shown in (A) for the investigation to assess the impact of whey protein isolate (WPI) relative to casein (CAS) in virgin females (study 1), virgin males (study 2), reproductive females, spanning virgin, pregnant and lactating states (study 3), and their corresponding offspring (study 4). In study 4, all offspring were given a diet enriched with CAS regardless of their mothers’ diet. In study 1 and 2, virgin females (study 1) and males (study 2) were provided with diets enriched with CAS (*n* = 8 each study) or WPI (*n* = 10 each study) for 11 weeks (WK). In study 3, virgin females mice were fed CAS (*n* = 4) or WPI (n = 6) enriched diet for the first 5 weeks and thereafter made pregnant and consumed the respective diets until late lactation. The offspring born to the mothers were used in study 4, which contained male offspring (*n* = 7) of mothers fed CAS, female offspring (*n* = 10) of mothers fed CAS, male offspring (*n* = 12) of mothers fed WPI, and female offspring (*n* = 13) of mothers fed WPI. The impact of WPI relative to CAS on body weight is shown for (B) virgin and reproductive females and (C) the offspring. Data represent mean ± SEM of all the animals in each group, which were used as independent biological replicates. Data related to study 1 and 3 were analyzed together, while groups in study 4 were analyzed together, each by two‐way repeated‐measures ANOVA.

Much of the evidence of how whey proteins affect physiology and metabolic health including the impact on vWAT and sWAT have come from studies using male rodents, and in human studies, there has been a greater focus to use both sexes together in each investigation.^[^ ^7^ ^]^ As a way to gain knowledge of how females respond to dietary whey proteins, which would be important to know when developing dietary interventions for both sexes, in the present study, we compared the effect of whey proteins on the growth rate (body weight gain) of male and female mice and the accompanying impact on the gut metabolome (study 1 and 2 in Figure 1A). The experiment setup also allowed us to investigate for an impact of protein quality (whey proteins versus casein) within a fixed quantity of dietary carbohydrates and fat to selectively reduce the difference in vWAT and sWAT between males and females.^[^ ^16^, ^17^, ^18^, ^19^ ^]^ Since there is a strong (>75%) genetic contribution to the variance in body mass index and body fat distribution in humans and animals, which are passed from the mother to the offspring,^[^ ^20^, ^21^, ^22^ ^]^ we included reproductive females in the experimental setup to determine if any effects on body weight and the adiposity seen in the parent generation can be passed to the offspring of both sexes or to one sex (study 3 and 4 in Figure 1A). The study utilized a diet high in carbohydrate and low in fat (high C/F ratio) in combination with dietary whey protein isolate (WPI) to avoid the confounding effects related to high fat feeding.^[^ ^23^ ^]^ The controls were fed a casein (CAS) to keep consistency with our previously work, which showed that mice respond to CAS intake by gaining weight and increasing adiposity, and that these changes were driven by the gut microbiota and the gut metabolome.^[^ ^5^, ^10^ ^]^ Notably, replacing CAS with WPI reversed the effects^[^ ^5^, ^8^, ^11^, ^12^, ^24^, ^25^ ^]^ including which adipose tissues (vWAT or sWAT) store excess energy.^[^ ^7^ ^]^ Four studies were performed investigating the impact of protein quality on virgin females (study 1), virgin males (study 2), and reproductive females, spanning virgin, pregnant, and lactating stages (study 3). To assess how the quality of the protein in the mother's diet affects the offspring, the animals born to mothers fed WPI and CAS were both fed the same control (CAS enriched) diet at weaning (study 4; Figure 1A).

## 2 Experimental Section

**Animals**: The study was approved by the Animal Experimentation Ethics Committee in the University College Cork, Ireland (2018/022), and was licenced under the European Directive 2010/63/EU. Thirty C57BL/6J female mice and 36 male mice were purchased (Harlan; UK), at 4–5 weeks of age and were housed in pairs in individually ventilated cages (Allentown Europe), with bedding. A tunnel and nesting material were provided as enrichment (LBS, UK). The animals were maintained at 21–23 °C, within 12 h light/dark cycles. The mice had ad libitum access to food and water throughout the study unless otherwise stated. In each of the studies, mice were provided with a diet containing 20% CAS and 70% carbohydrate (#D12450B; Research Diets, USA; % values by energy; Table ST1, Supporting Information) during the initial first week of the acclimatization period. Subsequently, at the age of approximately 6 weeks, weight matched mice either continued to consume the diet enriched with CAS or were switched to 20% WPI with 70% carbohydrate (Table ST1, Supporting Information). In study 1 and 2, virgin females (study 1) and males (study 2) were provided with diets enriched with CAS (*n* = 8 in each study) or WPI (*n* = 10 in each study) for 11 weeks (Figure 1A). In study 3, the virgin female mice fed CAS (*n* = 4) or WPI (*n* = 6) upon reaching sexual maturity, at the age of 12 weeks, were made pregnant by individually pairing with a single male mice that came from a different cohort to that used in study 2 (Figure 1A). The females that were presumed to be pregnant and had a history of co‐habiting (when they were virgins) were re‐housed together, and continued to consume the same diet during pregnancy and after giving birth as well as into the lactation period (Figure 1A). Each mother fed either WPI or CAS had on average four suckling pups. When the offspring were 4 weeks of age, and regardless of the history of the mother's diet (CAS or WPI), all offspring were given the CAS enriched diet for further 4 weeks (Study 4 in Figure 1A). At the end of each study, mice were fasted for 12 h, anesthetized by subcutaneous injection of 100 mg kg^−1^ Ketamine and 1 mg kg^−1^ Medetomidine, and sacrificed. Trunk blood was collected into EDTA‐coated tubes, and plasma extracted. The tissues were harvested and weighed, stored at −80 °C and along with the plasma for subsequent analysis.

**Metabolomics**: Two methods were used to ascertain the impact on the metabolome in the caecum and plasma (method 1) and in tissues (method 2). These methods were detailed in Nychyk et al.^[^ ^5^ ^]^ Briefly, in the first method, the cecal extracts and plasma corresponding to the reproductive females (study 3) and their respective virgin controls (study 1), as well as the offspring (study 4) were derivatized with methyl chloroformate and analyzed using gas chromatography (7890B, Agilent) coupled with a quadropole mass spectrometry detector (5977B, Agilent). The system was controlled by ChemStation (Agilent). Raw data were converted to netCDF format using Chemstation (Agilent), before the data was imported and processed in Matlab R2018b (Mathworks, Inc.) using the PARADISe software described by Johnsen et al.^[^ ^26^ ^]^ This analysis included 19 amino acids, 5 metabolites related to carbohydrates, 6 short chain fatty acids (SCFA), and 9 medium to long chain fatty acids. In the second method, the extracts of the grounded colon and duodenal tissues in cold extraction solvent (Ethanol: Phosphate Buffered Saline, 85:15), were mixed with 50 µL of 5% phenyl isothiocyanate derivatization solution in ethanol/water/pyridine (volume ratio 1/1/1), and then with 300 µL 5 mM ammonium acetate in methanol. The samples were subsequently centrifuged at 500 × *g* for 2 min, and the elute diluted 1:1 with HPLC‐grade water for LC–MS/MS analysis or diluted 1:10 with mobile phase for tandem mass spectrometry (FIA‐MS/MS) analysis. The samples were run on a SCIEX QTRAP 6500plus mass spectrometer coupled to SCIEX ExionLC Series UHPLC system. The analysis included 21 amino acids and 21 biogenic amines as well as 40 acylcarnitines, 14 lysophosphatidylcholines (lysoPCs), 76 phosphatidylcholines (PCs), 15 sphingomyelins (SMs), and the sum of hexoses (H1). The multiple reaction monitoring (MRM) method was used to quantify or semi‐quantify metabolites and data were acquired by AB Sciex Analyst version 1.7.2 software.

**Hormone and Glucose Measurements**: Plasma levels of insulin‐like growth factor (IGF)‐1, insulin, and glucose were measured using commercially available kits and according to the manufacturer's instructions (Mouse Glucose Assay Kit, CrystalChem, USA; Ultrasensitive Mouse Insulin ELISA kit; CrystalChem, USA; IGF‐1 ELISA kit, R&D systems, UK).

**RNAseq**: The related work was undertaken from archived tissues from the previous study,^[^ ^8^ ^]^ wherein, and similar to the present study, mice were fed 20% energy WPI with 70% energy carbohydrates (referred as WPI‐HS in Nilaweera et al.^[^ ^8^ ^]^), while the control group received a diet enriched with CAS (referred as CAS‐HS). As detailed previously,^[^ ^5^ ^]^ total RNA was extracted from the ileum and hypothalamic blocks and were paired‐end (PE) sequenced by PE100 on BGISEQ‐500 platform at Beijing Genomic Institute (PE 2 × 75 bp, 150 bp per fragment, 20 M read‐pairs per sample). The raw sequence reads, obtained in FASTQ format, were assessed for quality, trimmed, and cleaned of adaptor sequences using BBDuk java package. The trimmed reads were aligned to the *Mus musculus* reference genome assembly GRCm38 using STAR RNAseq aligner v2.5.2, and uniquely mapped read counts per Ensembl annotated gene/transcript were estimated using the STAR–quantMode option. Differential gene expression and data transformations and visualization were carried out using DeSeq2 v1.18.1 in R 3.5.2. The Ingenuity Pathway Analysis (IPA) program and the IPA Knowledge Database were used to assess the impact of the diet on the metabolic pathways regulated by different genes (Ingenuity Systems; https://www.qiagenbioinformatics.com).

## 3 Statistical Analyses

The data were analyzed using SPSS software version 24 (IBM Corp), SigmaPlot software version 15 (Inpixon, USA), and R statistical programming language (R Core Team, 2023). The normality and equal variance across data sets were established using Levene's, Shapiro‐Wilk, and/or Brown–Forsythe tests. Where appropriate, the data were log transformed to establish normality. The data related to body weight and food intake were analyzed separately by a two‐way repeated‐measures ANOVA to determine the main effect of protein type and time, as well as their interaction. The assumption of sphericity was tested using Mauchly's test, and the Greenhouse–Geisser correction was applied where necessary. The tissue weights, plasma hormones, and metabolites were analyzed by Univariate factorial ANOVA with post hoc analysis undertaken with Bonferroni to determine differences between groups. Data related to the pup weights and metabolites in the colon and duodenum were analyzed by unpaired *t*‐test. Data are presented as mean ± SEM. Pearson correlation was applied to find significant interacts between cecal metabolites and growth related parameters of the offspring. For metabolomics and RNAseq data, *p*‐values were adjusted for multiple comparisons using a Benjamini and Hochberg (B–H) method. A significance level of 0.05 was considered for all statistical tests.

## 4 Results

### 4.1 WPI Reduced the Body Weight Gain and Food Intake in Virgin Females, but Not in Virgin Males

There was no significant effect of protein quality on the body weight trajectory of virgin females (Figure 1B). The food intake decreased in virgin females fed WPI (2.24 ± 0.02 g in WPI fed vs 2.41 ± 0.04 g in CAS fed per mouse per day; protein effect, *p* = 0.006; time effect, *p* < 0.001). Interestingly, there was an interaction between the protein quality and time on food intake (*p* = 0.005), largely during week 6–11. Although the reduction in food intake was not reflected in the body weight, the cumulative gain in body weight of virgin females fed WPI (3.84 ± 0.37 g) showed a trend towards a decrease compared to the controls fed CAS (5.2 ± 0.62g ) over the 11 weeks (*p* = 0.069). Focusing specifically on weeks 6–11, the cumulative weight gain in virgin females fed WPI (1.33 ± 0.3 g) reduced compared to the controls (2.38 ± 0.32 g) (*p* = 0.035). The sensitivity of the female mice to whey proteins specifically during weeks 6–11 of diet intake, which corresponds to 13–18 weeks of age, remains to be determined. It is interesting that this period falls within adulthood, which they attain by age around 9.5 weeks.

Intake of WPI had no significant impact on the body weight trajectory of males (27.54 ± 0.58 g on average) compared to controls fed CAS (27.42 ± 0.58 g on average) (protein effect, *p* = 0.89; time effect, *p* < 0.0001; time × protein effect, *p* = 0.10). The quality of protein had no significant effect on the overall food intake over 11 weeks (3.33 ± 0.12 g in WPI fed vs 3.07 ± 0.13 g in CAS fed per mouse per day; protein effect, *p* = 0.19; time effect, *p* = 0.008). However, over time, the intake of males fed WPI increased above controls fed CAS (time x protein effect, *p* = 0.046). The higher intake but an unaltered body weight, suggesting an energy loss, was also previously demonstrated by us using male mice fed WPI, where the animals eventually reduced weight gain by the end of the extended (17 weeks) study period.^[^ ^8^ ^]^ A similar energy loss has also been reported in humans fed WPI.^[^ ^7^ ^]^

### 4.2 WPI Reduced the Body Weight in Reproductive Females

The reproductive females fed WPI had a decreased body weight compared to the reproductive females fed CAS (Figure 1B). The effect was largely observant during pregnancy (*p* = 0.022), whereas there was only a trend towards a decrease in body weight during virgin (*p* = 0.098) and lactating (*p* = 0.063) stages. The food intake did not show a significant change in response to the quality of protein during the reproductive stages (data not shown; *p* = 0.13), albeit it increased, as expected, with the change in reproduction (data not shown; *p* < 0.001).

### 4.3 Offspring of Mothers Fed WPI Gained Less Body Weight

At late lactation (day 21), suckling pups of mothers fed WPI had a reduced body weight (6.83 ± 0.17 g ) compared to those nursed by mothers fed CAS (8.69 ± 0.32g ; *p* < 0.001). Although all offspring were weaned into a CAS enriched diet, the history of the mother's diet continued to impact on the growth of the offspring across both sex (Figure 1C). This effect was not due to changes in food intake (male offspring of mothers fed WPI, 3.01 ± 0.08 g and female offspring of mothers fed WPI, 2.64 ± 0.05 g vs male offspring of mothers fed CAS, 3.10 ± 0.05 g and female offspring of mothers fed CAS, 2.67 ± 0.04 g per mouse per day; *p* = 0.38), although the expected difference in intake between males and females was observed (*p* < 0.001) and with time (*p* < 0.001).

### 4.4 WPI Intake Reduced Adiposity across Generations

The virgin females that continued to consume WPI over the 11 weeks had reduced gonadal white adipose tissue (gWAT) and retroperitoneal white adipose tissue (rWAT) and there was a trend towards a decrease in interscapular brown adipose tissue (iBAT; *p* = 0.068) and subcutaneous white adipose tissue (sWAT; *p* = 0.091) compared to virgin females fed CAS (Table 1). While feeding of WPI up to lactation stage continued to reduce gWAT and iBAT, the effect seen in virgin females on the rWAT was lost by late lactation, while sWAT showed increased weight in lactating females fed WPI compared to lactating controls fed CAS (Table 1). Despite the remodeling of the adipose tissues in lactating females fed WPI, in the next generation, female offspring born to mothers fed WPI had reduced sWAT and gWAT (Table 2), similar to the virgin females in the previous generation (Table 1). In contrast, the male offspring born to mothers fed WPI only had reduced gWAT compared to male counterparts of mothers fed CAS (Table 2).

**TABLE 1:** The impact of whey protein isolate (WPI) or casein (CAS) on the weight of tissues in virgin or lactating females.

Tissue	Virgin CAS	Virgin WPI	Lactating CAS	Lactating WPI	Significance at *p* < 0.05		
					Prot:	Repro:	Inter:
Colon	114.4 ± 3.6	103.9 ± 4.8	153.0 ± 12.4**^a^**	112.6 ± 2.6**^b^**	<0.001	<0.001	0.022
Intestine	618.7 ± 23.3**^c^**	529.1 ± 11.6**^d^**	789.4 ± 42.3	750.1 ± 43.1	0.056	<0.001	0.43
gWAT	316.2 ± 45.7	217.2 ± 21.3	299.1 ± 44.9	170.3 ± 17	0.002	0.32	0.64
sWAT	460.6 ± 88.4	274.4 ± 26.3	612.9 ± 89.2 **^e^**	918.6 ± 98.1 **^f^**	0.45	<0.001	0.005
rWAT	164.7 ± 34.8**^g^**	107.9 ± 8.3**^h^**	114.8 ± 21.7	72.4 ± 5.9	0.017	0.037	0.70
iBAT	81.3 ± 5.4	68.4 ± 2.2	116.8 ± 10.7**^i^**	86.5 ± 5.7**^j^**	0.002	<0.001	0.15
Liver	895.8 ± 50.4	802.5 ± 17.8	1647.0 ± 92.0**^k^**	2154.3 ± 111.1**^l^**	0.013	<0.001	<0.001
Pancreas	174.5 ± 14.6	164.0 ± 9.3	207.4 ± 18.9	178.6 ± 16.3	0.19	0.12	0.54

The significance of date (mean ± SE in mg) is shown with regard to the effect of protein quality (Pro:), reproduction (Repro:) and their interactions (inter;). Data analyzed by Univariate factorial ANOVA with post hoc analysis undertaken with Bonferroni. Within the virgin group fed CAS (*n* = 8) or WPI (*n* = 10) or within the lactating group fed CAS (*n* = 4) or WPI (*n* = 6), the significance is shown as ^a^ versus ^b^ (*p* < 0.001), ^c^ versus ^d^ (*p* = 0.049), ^e^ versus ^f^ (*p* = 0.016), ^g^ versus ^h^ (*p* = 0.039), ^i^ versus ^j^ (*p* = 0.002), ^k^ versus ^l^ (*p* < 0.001). Gonadal white adipose tissue (gWAT), subcutaneous WAT (sWAT), retroperitoneal WAT (rWAT), and interscapular brown adipose tissue (iBAT).

**TABLE 2:** The weight of tissues of male and female offspring of mothers fed whey protein isolate (WPI) or casein (CAS).

Tissue	Male off Moth‐CAS	Male off Moth‐WPI	Female off Moth.‐CAS	Female off Moth.‐WPI	Significance at *p* < 0.05		
					Prot:	Sex	Inter:
Colon	100.6 ± 4.7**^m^**	84.0 ± 2.9**^n^**	83.4 ± 2.2	83.5 ± 2.4	0.011	0.006	0.009
Intestine	577.2 ± 13.6**^0^**	503.2 ± 15.5**^p^**	411.2 ± 20.3	431.5 ± 22.7	0.18	<0.001	0.024
gWAT	342.7 ± 19.6	286.8 ± 30.2	254.3 ± 19.2	209.7 ± 13.2	0.036	<0.001	0.80
sWAT	291.7 ± 41.2	319.2 ± 23.2	454.6 ± 26.8**^q^**	374.2 ± 17.4^r^	0.32	<0.001	0.046
rWAT	86.6 ± 10.0	85.5 ± 12.6	164.9 ± 13.8	149.0 ± 10.4	0.50	<0.001	0.56
iBAT	96.8 ± 8.9	95.8 ± 6.4	98.2 ± 8.0	97.6 ± 6.9	0.91	0.83	0.98
Liver	768.8 ± 51.3	716.1 ± 45.7	637.8 ± 22.0	579.1 ± 38.6	0.18	0.003	0.94
Pancreas	196.6 ± 8.5	178.4 ± 10.1	148.2 ± 12.1	151.1 ± 10.5	0.49	0.002	0.34

The significance of date (mean ± SE in mg) is shown on offspring (off) of mothers (Moth) fed WPI or CAS with regard to protein quality (Pro:), sex and their interactions (inter;). The studies contained male offspring (*n* = 7) of mothers fed CAS, female offspring (*n* = 10) of mothers fed CAS, male offspring (*n* = 12) of mothers fed WPI and female offspring (*n* = 13) of mothers fed WPI. Data analyzed by Univariate factorial ANOVA with post hoc analysis undertaken with Bonferroni. Within the male and females groups, the significance is shown as ^m^ versus ^n^ (*p* < 0.001), ° versus ^p^ (*p* = 0.018), ^q^ versus ^r^ (*p* = 0.027). Gonadal white adipose tissue (gWAT), subcutaneous WAT (sWAT), retroperitoneal WAT (rWAT), and interscapular brown adipose tissue (iBAT).

### 4.5 WPI Affected the Cecal Metabolome in Virgin and Lactating Females and Their Offspring

**Figure 2:**
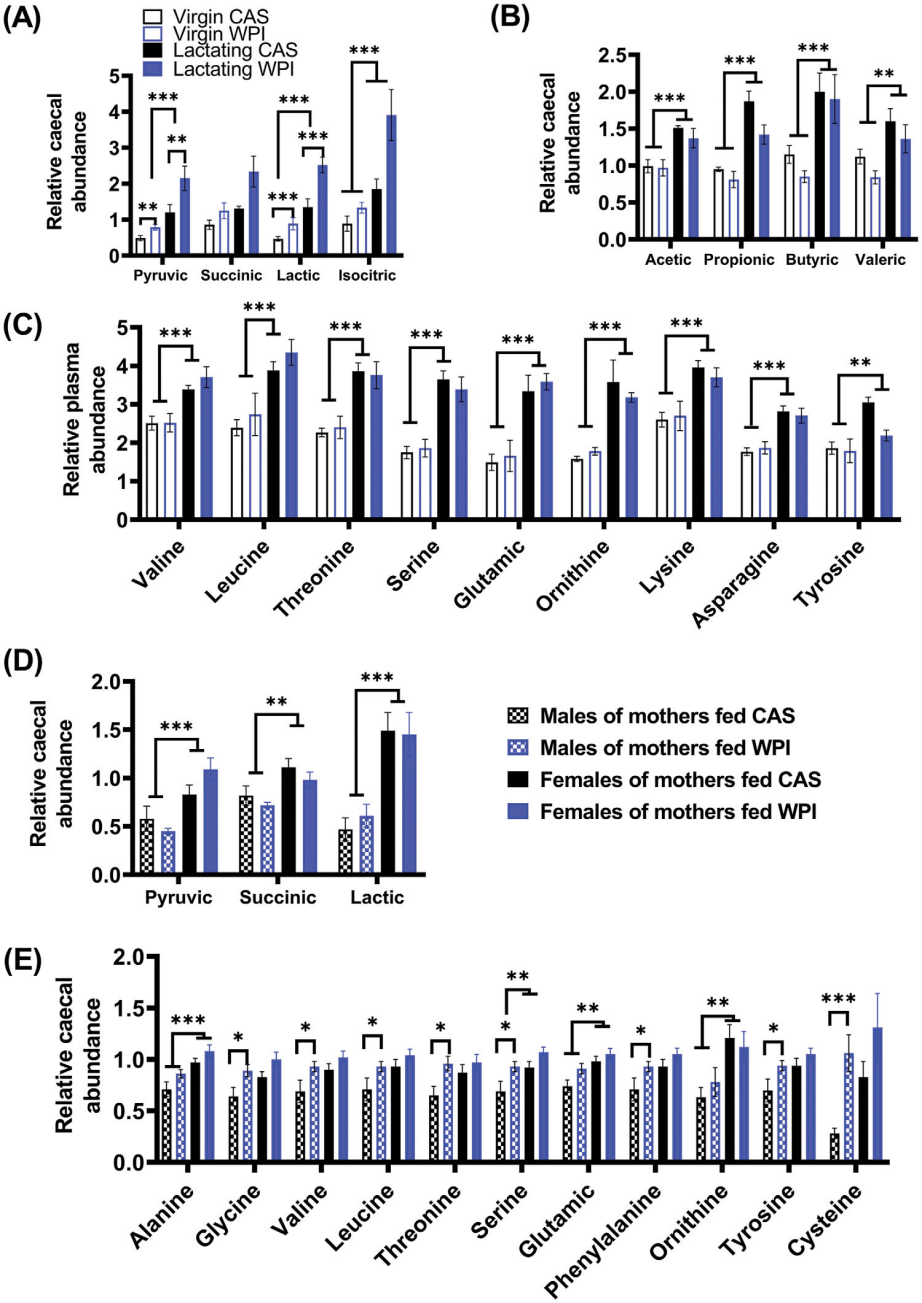
The impact of whey protein isolate (WPI) relative to casein (CAS) on the levels of cecal and plasma metabolites. The data relates to (A–C) virgin and lactating females and (D and E) the offspring. The levels are shown (A, B, D, and E) in the cecum and (C) in the plasma. The studies contained virgin female mice fed CAS (*n* = 8) or WPI (*n* = 10), lactating female fed CAS (*n* = 4) or WPI (*n* = 8), male offspring (*n* = 7) of mothers fed CAS, female offspring (*n* = 10) of mothers fed CAS, male offspring (*n* = 12) of mothers fed WPI and female offspring (*n* = 13) of mothers fed WPI. Data represent mean ± SEM of all the animals in each group, which were used as independent biological replicates. Data related to study 1 and 3 were analyzed together, while groups in study 4 were analyzed together by Univariate factorial ANOVA with Bonferroni used for post hoc analysis. *p < 0.05; **p < 0.005; ***p < 0.001.

Despite the reduction in food intake, cumulative weight gain and the intestinal weight in virgin females fed WPI (Table 1), the metabolome of the duodenum and the colon was unaffected (Table S2, Supporting Information). However in the cecum, the relative levels of pyruvic and lactic acids increased in virgin females fed WPI compared to controls fed CAS (FDR < 0.05; Figure 2A). Superimposing the effect of lactation coupled with WPI intake further increased the level of these metabolites (FDR < 0.05: Figure 2A). Intake of WPI had no significant impact on cecal level of amino acids (Table S3, Supporting Information) or on lactation‐induced increased cecal level of SCFA (FDR < 0.05; Figure 2B) and plasma amino acids (FDR < 0.05: Figure 2C).

Similar to the lactating females, their female offspring had an increased cecal level of pyruvic, succinic, and lactic acid compared to male offspring (FDR < 0.05: Figure 2D). In addition, the cecal availability of alanine and glutamic acid was increased in the female compared to male offspring (FDR < 0.05: Figure 2E), while serine and ornithine showed similar trends (Figure 2E; serine, FDR = 0.07 and ornithine, FDR = 0.052). Interestingly, while the quality of protein in the mother's diet had no effect on metabolites related to carbohydrate metabolism in the cecal contents of both sexes (Figure 2D), the cecal level of cysteine showed a trend towards increased in male and female offspring (*p* = 0.002; FDR = 0.084) born to mothers fed WPI (Figure 2E). When the effect was separated by sex, the greatest increase was seen in male offspring (FDR < 0.05: Figure 2E). Similarly, glycine, valine, leucine, threonine, serine, phenylalanine, and tyrosine were significantly increased in the cecum of male offspring born to mothers fed WPI (Figure 2E). In contrast to the cecal contents, neither the sex of the offspring nor the quality of protein in the mother's diet had any impact on the plasma level of metabolites in the offspring (Table S4, Supporting Information).

### 4.6 Intake of WPI Reduced Plasma Glucose Levels in Lactating Females

**Figure 3:**
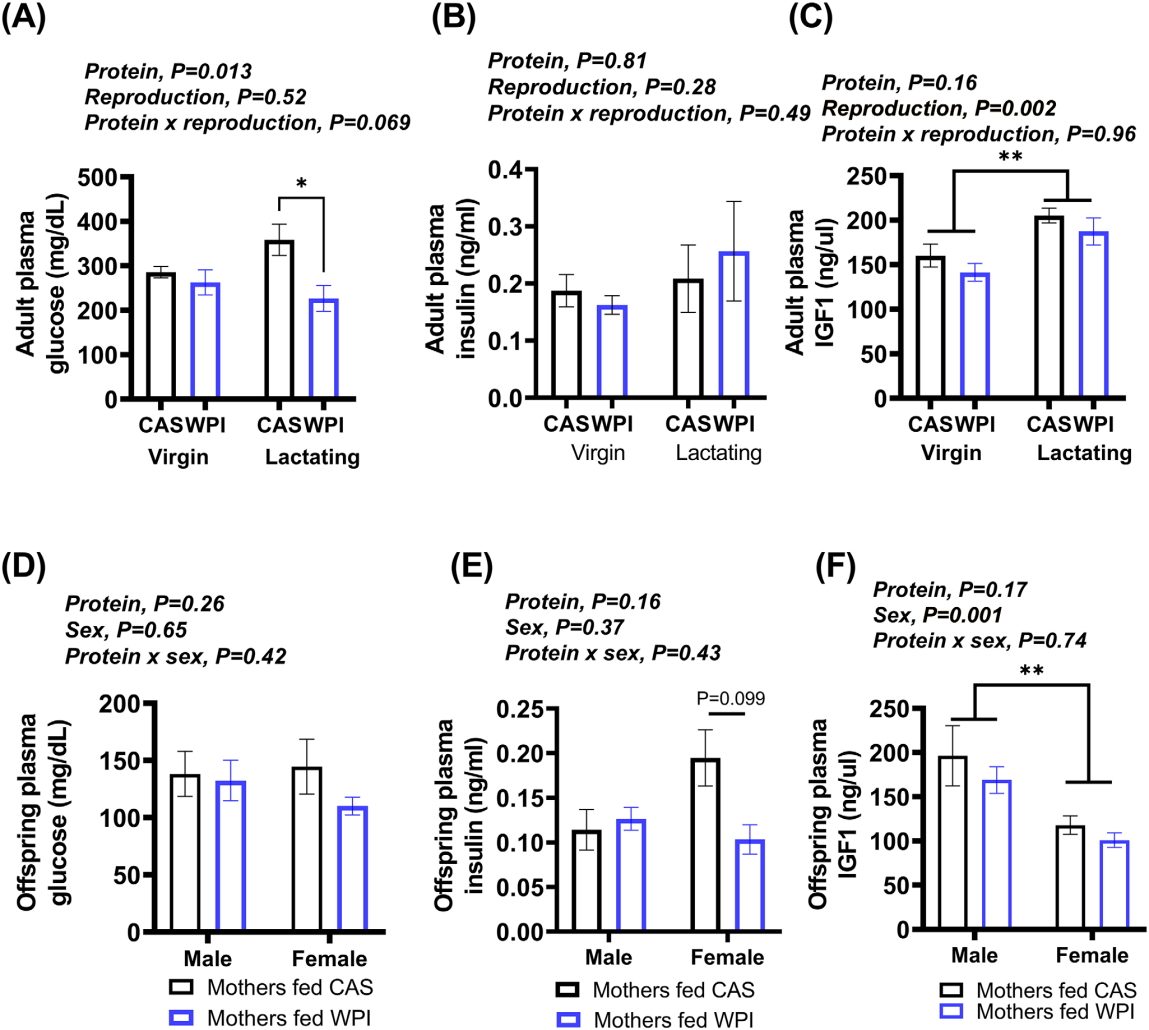
The impact of whey protein isolate (WPI) relative to casein (CAS) on the plasma availability of glucose and hormones. Shown are the levels of (A and D) glucose, (B and E) insulin, and (C and F) insulin‐like growth factor (IGF)1 in (A–C) adult virgin and lactating females and (D–F) in the offspring. The studies contained virgin female mice fed CAS (*n* = 8) or WPI (*n* = 10), lactating female fed CAS (*n* = 4) or WPI (*n* = 8), male offspring (*n* = 7) of mothers fed CAS, female offspring (*n* = 10) of mothers fed CAS, male offspring (*n* = 12) of mothers fed WPI and female offspring (*n* = 13) of mothers fed WPI. Data represent mean ± SEM of all the animals in each group, which were used as independent biological replicates. Data related to study 1 and 3 were analyzed together, while groups in study 4 were analyzed together by Univariate factorial ANOVA with Bonferroni used for post hoc analysis *p < 0.05; **p < 0.005.

The plasma level of glucose was reduced in lactating females fed WPI compared to the controls fed CAS (Figure 3A). The quality of the protein had no effect on plasma insulin levels or on insulin‐like growth factor (IGF‐1)(Figure 3B,C), albeit, IGF‐1 levels responded to the change in the reproductive status (Figure 3C). The quality of the protein in the mother's diet had no significant impact on the plasma glucose, insulin or IGF‐1 levels in the offspring (Figure 3D–F), albeit the expected difference in IGF‐1 level was seen between males and females (Figure 3F).

### 4.7 Correlations Exist between Cecal Metabolites, IGF1, and Adiposity in the Offspring

**Figure 4:**
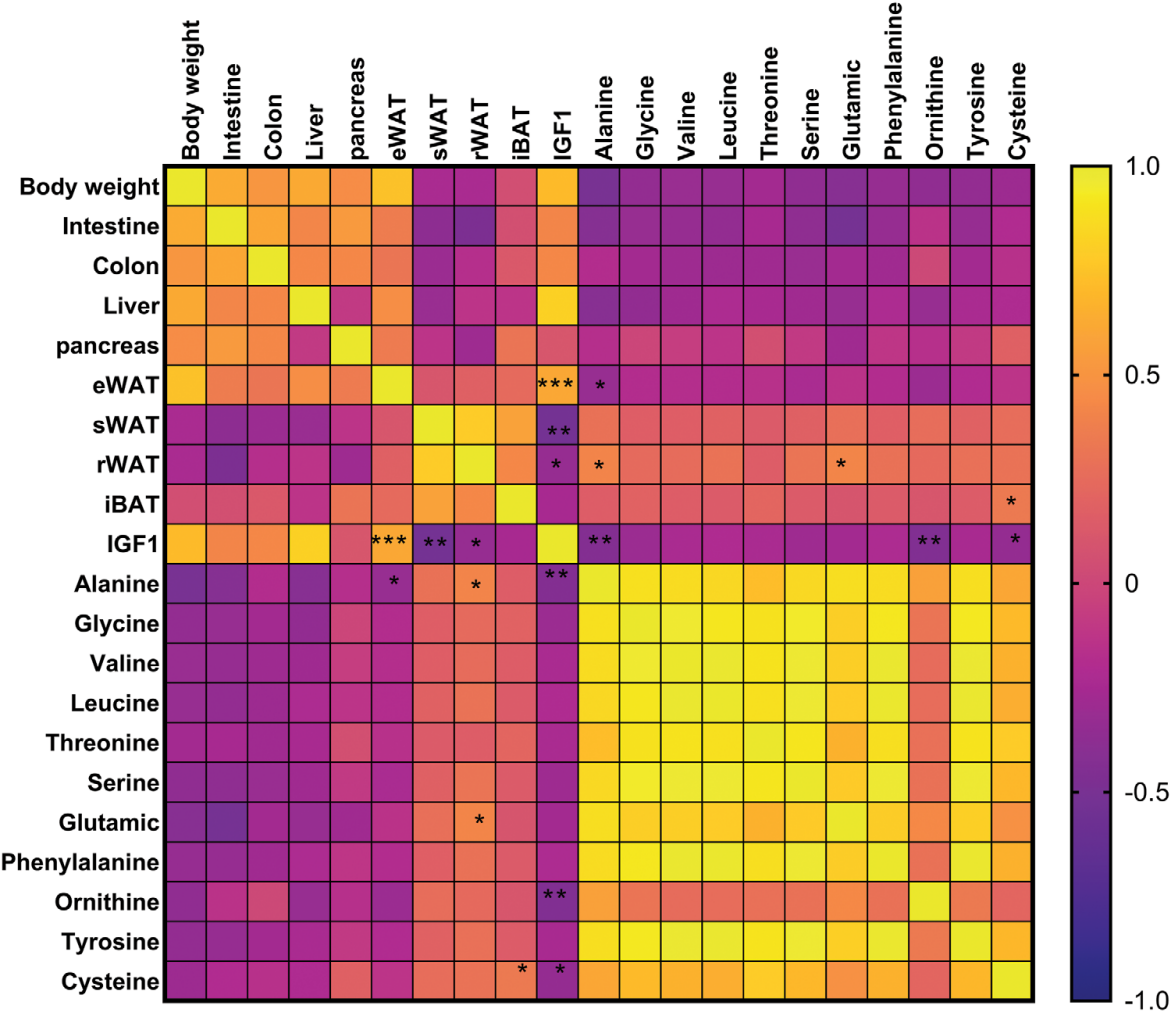
Correlation between cecal metabolites and growth related parameters in the offspring. The significance of the Pearson correlation is highlighted specifically between cecal amino acids, insulin like growth factor (IGF) 1 and the weight of adipose tissues for male offspring (*n* = 7) of mothers fed CAS, female offspring (*n* = 10) of mothers fed CAS, male offspring (*n* = 12) of mothers fed WPI, and female offspring (*n* = 13) of mothers fed WPI. The significance of other interactions are shown in Table S6, Supporting Information. *p < 0.05; **p < 0.005, ***p < 0.001.

In searching for associations between gut metabolome and growth, it was notable that IGF1 was positively correlated with eWAT and negatively correlated with the sWAT and rWAT. In contrast, the cecal abundance of alanine and ornithine, two amino acids found to be higher in female compared to male offspring (Figure 2E), as well as cysteine, an amino acid higher in male offspring born to mothers fed WPI (Figure 2E), were found to be negatively correlated with plasma IGF1 levels (Figure 4). The complexity of the potential interaction between gut activity and adiposity is further highlighted by the positive correlation between cecal abundance of alanine and rWAT and the negative correlations between alanine and eWAT (Figure 4). No such correlation existed for ornithine with adipose tissues, whereas cysteine was positively correlated with iBAT (Figure 4). All significant correlations not listed above are detailed in Table S5, Supporting Information.

### 4.8 Long Term Intake of WPI Affected the Ileal and Hypothalamic Gene Expression in Virgin Males

**Figure 5:**
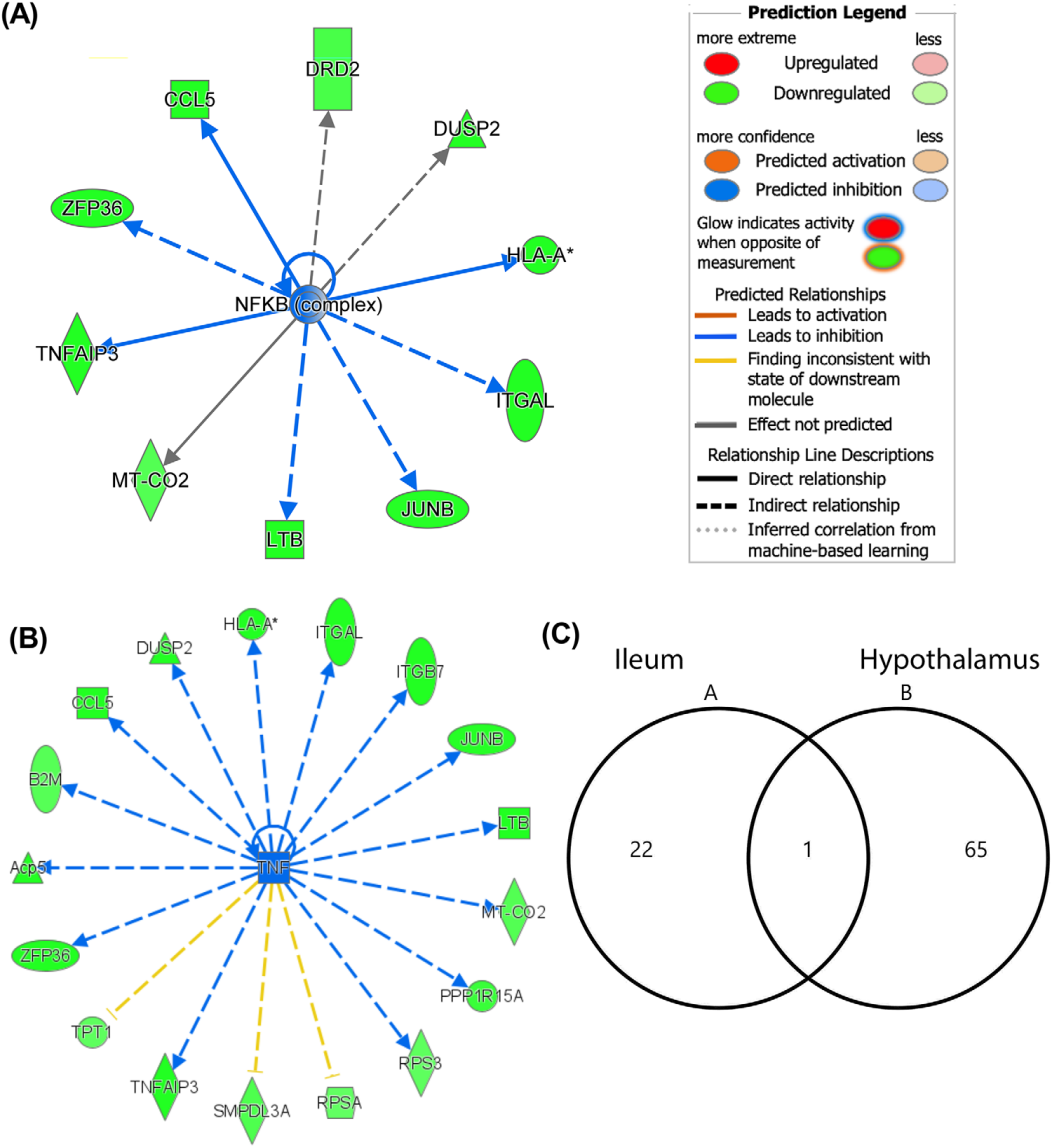
The clausal networks associated with the hypothalamic transcriptome responding to whey protein isolate (WPI) relative to casein (CAS). The impact is shown on the (A) nuclear factor‐kappa B (NFκB) and (B) tumor necrosis factor (TNF). The Venn diagram in (C) shows the number of distinct genes and the only common gene responding to WPI relative to CAS in the ileum and hypothalamus. The data related to the transcriptome were generated from archived tissues of male mice fed WPI or CAS for 17 weeks.^[8]^ The gene abbreviations can be found in Table 3.

To understand the cellular response in virgin males fed WPI, the ileal transcriptome was analyzed in archived tissues of a previous published study, where virgin males were fed WPI or CAS for an extended (17 week) period.^[^ ^8^ ^]^ Data revealed 23 differentially expressed genes in the ileum, which can be broadly categorized into having roles in growth/energy balance and immunity (Table 3). The genes in the first category responded to WPI intake by reducing the expression of fibroblast growth fact (FGF)15, neuropeptide Y (NPY), vasoactive intestinal peptide (VIP), and phospholipase B1, while the expression of beta‐carotene oxygenase 1 and chymotrypsinogen B1 (CTRB1) increased (Table 3). In parallel, the genes affecting cell structure showed an increased expression, namely for eukaryotic translation initiation factor 2 alpha kinase 3 (EIF2AK3), gap junction protein beta 4 (GJB4), and 3“‐phosphoadenosine 5”‐phosphosulfate synthase 1 (PAPSS1) (Table 3). Accompanying the latter changes, the genes related to immunity increased (metallothionein 1), while lipocalin 2 and histocompatibity 2, Q region associated gene decreased in expression (Table 3). In contrast to the ileum, more hypothalamic genes responded (Table S6, Supporting Information). Pathway analysis predicted a down regulation of nuclear factor‐kappa B (NFκB) (*Z* score −2.56) (Figure 5A), tumor necrosis factor‐alpha (TNFα) (*Z* score −2.97) (Figure 5B) and signal transducer and activator of transcription 3 (STAT3; *Z* score −2.89). In addition to immune related genes, a number of genes encoding ribosomal proteins decreased in expression in response to WPI intake (Table S6, Supporting Information). Pathway analysis predicted a reduction in the quantity of cells in the tissue (*Z* score −2.16). Interestingly, the genes affected in the ileum were distinct to those affected in the hypothalamus, with only one common responsive gene, namely the histocompatibility locus associated gene (Figure 5C).

**TABLE 3:** The impact of whey protein isolate relative to casein on the ileal transcriptome of male mice.

Gene ID	Gene symbol	Gene name	*p*‐Adjusted	Fold change
ENSMUSG00000031957	CTRB1	Chymotrypsinogen B1	0.0405	48.05
ENSMUSG00000046623	GJB4	Gap junction protein beta 4	0.00102	4.05
ENSMUSG00000031668	EIF2AK3	Eukaryotic translation initiation factor 2 alpha kinase 3	0.00135	1.37
ENSMUSG00000028032	PAPSS1	3′‐phosphoadenosine 5′‐phosphosulfate synthase 1	0.0488	1.24
ENSMUSG00000002565	SCIN	Scinderin	0.0355	1.30
ENSMUSG00000031845	BCO1	Beta‐carotene oxygenase 1	0.00105	1.92
ENSMUSG00000028962	SLC4A2	Solute carrier family 4 member 2	0.00482	1.30
ENSMUSG00000031765	Mt1	Metallothionein 1	0.00675	1.56
ENSMUSG00000005125	NDRG1	N‐myc downstream regulated 1	0.0375	1.35
ENSMUSG00000022453	NAGA	Alpha‐N‐acetylgalactosaminidase	0.007	1.20
ENSMUSG00000031613	HPGD	15‐hydroxyprostaglandin dehydrogenase	0.0375	1.27
ENSMUSG00000029134	PLB1	Phospholipase B1	4.03E‐07	−115.38
ENSMUSG00000046697	ENPP7	Ectonucleotide pyrophosphatase/phosphodiesterase 7	0.00101	−17.16
ENSMUSG00000091721	GIMD1	GIMAP family P‐loop NTPase domain containing 1	0.007	−11.48
ENSMUSG00000047502	Mroh7	Maestro heat‐like repeat‐containing protein family member 7	0.033207	−5.25
ENSMUSG00000031073	FGF15	Fibroblast growth factor 15	0.0376	−3.77
ENSMUSG00000029819	NPY	Neuropeptide Y	0.02	−2.84
ENSMUSG00000026614	SLC30A10	Solute carrier family 30 member 10	0.000168	−2.13
ENSMUSG00000055730	Ces2a	Carboxylesterase 2A	1.88E‐05	−1.74
ENSMUSG00000031298	ADGRG2	Adhesion G protein‐coupled receptor G2	0.00482	−2.75
ENSMUSG00000026822	LCN2	Lipocalin 2	0.00102	−2.75
ENSMUSG00000019772	VIP	Vasoactive intestinal peptide	0.0405	−1.65
ENSMUSG00000091705	H2‐Q2	Histocompatibility 2, Q region locus 2	0.0332	−1.45

The data were generated following application of RNAseq on archived tissues from a previously published study involving male mice fed whey proteins or casein (*n* = 8).^[^ ^8^ ^]^ Positive numbers refer to increased and negative numbers refer to decreased expression. P values were adjusted for multiple comparisons using a Benjamini and Hochberg (B–H) method.

## 5 Discussion

The data show that female virgin mice are more responsive to WPI than male mice, wherein the males required a period of intake (17 weeks) beyond what was provided in the present study (11 weeks) to decrease body weight gain and gWAT, which was demonstrated previously by us.^[^ ^8^ ^]^ In the responsive females, WPI reduced the vWAT (gWAT and rWAT), while a modest effect can be seen in the sWAT and iBAT. This contrasts with the less responsive male mice that only decreased gWAT in the vWAT compartment after prolong (17 weeks) intake.^[^ ^8^ ^]^ The data suggested a sex difference in the response of the adipose tissues and growth (body weight gain) to WPI intake relative to CAS. In the search for the mechanisms, our previous published study^[^ ^8^ ^]^ showed an altered composition of the gut microbiota in male mice fed WPI, reducing the abundance of Firmicutes, which are known to have roles in energy harvest.^[^ ^27^ ^]^ This coupled with the increased energy content observed in the feces and reduced ileal expression of the gene for SLC6a19 involved in the transport of methionine, without a change in expression of the glucose transporter, SLGT1,^[^ ^8^ ^]^ suggested an impact of WPI on the breakdown of dietary proteins in the gut of male hosts.^[^ ^28^, ^29^ ^]^ In the more responsive virgin female mice fed WPI, the greatest impact was seen on the cecal abundance of pyruvic and lactic acid, which increased compared to CAS fed controls. These findings provided the first indication of an impact of WPI affecting the catabolism of dietary proteins (in males) and carbohydrates (in females) within the gut, which can potentially influence the animals’ growth trajectory and adiposity. For the responsive females, the effect seen perhaps relate to WPI‐induced enhancement of gut activity that we know to protect females from impairment in glucose metabolism.^[^ ^30^ ^]^

Superimposing the energy demand of reproduction,^[^ ^31^, ^32^, ^33^, ^34^ ^]^ further increased cecal abundance of metabolites of carbohydrate catabolism (pyruvic, isocitric, and lactic acids) as well as SCFA, known to be produced by saccharolytic fermentation.^[^ ^35^ ^]^ Changing the quality of the protein from casein to WPI in reproductive mothers, increased the cecal abundance of metabolites of carbohydrate catabolism above lactating mothers fed CAS, which were even higher than virgin controls. In this background, and with an unaltered SCFA and IGF1, which are known to affect energy balance and glucose homeostasis,^[^ ^36^, ^37^ ^]^ the mothers fed WPI showed re‐modeling of tissues and reduced plasma levels of glucose. The latter finding is significant given the link between high carbohydrate intake and metabolic disturbances that affect glucose homeostasis.^[^ ^38^, ^39^, ^40^ ^]^ We propose that the above outcomes reflect a change in energy partitioning in tissues, arising from the energy deficit created by the high carbohydrate catabolism in the gut of the mothers fed WPI. In line with this suggestion, the suckling pups of these mothers had a lower body weight at late lactation.

There is a large body of evidence suggesting that the maternal diet impacts on pre and post‐natal development and that the maternal gut microbiota manifests health outcomes in the offspring.^[^ ^41^, ^42^ ^]^ Our data show that there is a sex preference of WPI with regard to body weight in the parent generation (with females responding more), but not in the next generation, as both males and female offspring responded, without affecting the known sex‐difference in food intake, body weight, and circulating IGF1 levels.^[^ ^16^, ^43^ ^]^ Thus, we reasoned that some change must have occurred during the transmission of WPI effects across the generations. Interestingly, while the higher gut capacity to breakdown dietary carbohydrates seen in virgin females, which was enhanced in mothers fed WPI, manifested in the female offspring, in the same animals, a previously un‐noticed capacity to breakdown dietary proteins in the gut emerged, reflected by increased abundance of alanine, serine, glutamic acid, and ornithine in the cecal contents. The latter effects occurred regardless of the quality of the protein consumed by the mother. This contrast with the male offspring that responded to the quality of the protein in the mother's diet and retained the gut capacity to breakdown dietary proteins seen in the males of the parental generation with the extended WPI intake.^[^ ^8^ ^]^ The data provided an insight into how the impact of WPI within the gut of each sex gets modified when passed into the next generation. The finding of both positive and negative correlations between IGF1 and specific adipose compartments, and similar correlations with cecal amino acids, which were found to be higher in the female offspring (alanine, glutamic acid, and ornithine) or specifically higher in male offspring of mothers fed WPI (cysteine), suggest a complex route of how the transmitted gut activity can influence physiological outcomes in the host.

If the dietary protein catabolism in the gut is an important mechanism by which WPI mediates its effects on growth, as suggested in male mice in the two generations as well as in female offspring that responded equally to male counterparts in terms of body weight, then there should be some evidence of its impact reflected in the cells of the host. To test this, we focused attention on the ileal transcriptome in virgin males fed WPI that were previously shown to reduce body weight gain.^[^ ^8^ ^]^ In these animals, the ileal gene for FGF15 decreased in expression in line with a reduced dietary protein‐inputs into the cells,^[^ ^44^ ^]^ while the expression of CTRB1, involved in dietary protein hydrolysis, increased in expression by 48 fold. While these data support a greater dietary protein catabolism, it is unclear if the ileal expression of CTRB1 contributes to hydrolysis of peptides in the lumen or in the cells. This is because much of the luminal chymotrypsinogen is known to arise from the pancreatic production.^[^ ^45^ ^]^ Given the roles of FGF15 and NPY in growth and energy balance,^[^ ^44^, ^46^ ^]^ the corresponding reduction in expression of the genes provide a mechanistic understanding why males fed WPI had a reduced growth trajectory. In the ileum, this was also reflected in a change in cellular architecture, with effects on genes encoding GJB4 (with roles in gap junction),^[^ ^47^ ^]^ EIF2AK3 (which can inhibit translation),^[^ ^48^ ^]^ PAPSS1 (involved in sulfonation reactions),^[^ ^49^ ^]^ and VIP (affecting the formation of villi and crypts).^[^ ^50^ ^]^ Interestingly, the effect of WPI on the ileal transcriptome appears to be distinct to the hypothalamus, and yet, the related genes had similar roles, either related to growth/energy balance or in immunity. In the hypothalamus, the STAT3, which is important for growth and immunity,^[^ ^51^, ^52^, ^53^ ^]^ was predicted to be reduced in activity in male mice fed WPI. This finding also supports the predicted down‐regulation of NFκB and TNFα activity, as well as the expression of number of genes important for protein synthesis. We speculate that the tissue‐specific gene responses relate to the higher metabolites produced by the catabolism of dietary proteins in the gut of male mice fed WPI relative to CAS, which together act to reduce growth. We further speculate that the sex differences in the catabolism of dietary proteins (mainly in males) and carbohydrates (mainly in females) that arise more in the gut with WPI intake (relative to CAS) may also act with the sex differences in lipid metabolism^[^ ^54^, ^55^ ^]^ to cause a targeted reduction of vWAT in both sexes across generations and sWAT specifically in females, which was modest in the virgins in the first generation but accentuated in the virgin females in the next generations. We suspect these changes were driven by a unique interaction between WPI and high C/F ratio, which were distinct to the interaction between CAS and high C/F ratio because our previous work using the same diets showed that the specificity of the WPI to reduce the vWAT in male mice^[^ ^8^ ^]^ can be altered to reduce the sWAT simply by switching the C/F ratio from high (to deliver more carbohydrates) to low (to deliver more lipids).^[^ ^11^ ^]^ Adding antibiotics in drinking water disrupted the impact of the interaction between WPI and low C/F ratio on adiposity and gut metabolome, which were now similar to the antibiotic supplemented control group fed CAS with the same (low) C/F ratio‐enriched diet.^[^ ^10^ ^]^ This work, coupled with the correlations established in the current study between specific amino acids, IGF1, and adipose compartments suggest a potential functional relationship between gut activity and physiological outcomes, which appear to be driven by the interaction between dietary whey proteins and other macronutrients in the diet. The latter suggestion of an interaction between WPI and C/F ratio requires further experimental data to confirm.

## 6 Concluding Remarks

Intake of WPI, as part of a high carbohydrate diet, affected the growth and gut activity related to macronutrient catabolism in mice. The effect on growth showed a sex‐preference in the parent generation (more responsive in females), but not in the next generation. This appeared to be related to a sex‐difference in gut activity involved in the breakdown of dietary proteins (mainly in males) and carbohydrates (mainly in females) in the parent generation and to a change in the digestive characteristics when transmitted into the next generation, which allow male offspring to breakdown dietary proteins, while female offspring to breakdown both carbohydrates and proteins in the gut. The regulated transmission of male and female characteristics responding to WPI may arise because of specific interactions between gut metabolome and host genes in each sex.

## Conflict of Interest

The authors declare no conflict of interest.

## Author Contributions

Conceptualization (K.N.N.), data curation (K.N.N.), formal analysis (K.N.N., O.N., X.Y., P.C., A.S., L.B.), funding acquisition (D.B., K.N.N., J.R.S.), investigation (K.N.N., O.N., W.M., L.M., Q.A., D.P., J.D., X.Y., J.T.), methodology (K.N.N., X.Y., J.R.S., L.B., P.D.C.), supervision (K.N.N., J.T., J.R.S., D.B., L.B., P.D.C.), writing – original draft (K.N.N.), Writing – review and editing (all authors).

## Supporting information



Supporting information

Supporting information

Supporting information

Supporting information

Supporting information

Supporting information

## Data Availability

Data available on request from the authors. The RNAseq data will be made available in the European Genome‐Phenome Archive with accession number PRJEB77664.
