# Supplementary material for: The Sex Dependent and Independent Effects of Dietary Whey Proteins Are Passed from the Mother to the Offspring
Source: Mol Nutr Food Res. 2024 Nov 3;68(23):2400584. doi: 10.1002/mnfr.202400584 (PMC11653169; doi:10.1002/mnfr.202400584)
Supplement: Supplementary file 1 — Supporting information [file MNFR-68-2400584-s004.docx]

**Supplementary Table S1. Diet composition** ^a^

| Contents (g) | Diets | 1 |  | 2 |  |
| --- | --- | --- | --- | --- | --- |
|  |  | CAS enriched |  | WPI enriched |  |
| Casein |  | 200 |  | 0 |  |
| Whey protein isolate |  | 0 |  | 200 |  |
| L-Cystine |  | 3 |  | 3 |  |
| Corn Starch |  | 315 |  | 315 |  |
| Maltodextrin 10 |  | 35 |  | 35 |  |
| Sucrose |  | 350 |  | 350 |  |
| Cellulose, BW200 |  | 50 |  | 50 |  |
| Soybean oil |  | 25 |  | 25 |  |
| Lard |  | 20 |  | 20 |  |
| Mineral mix S10026A |  | 10 |  | 10 |  |
| Ca_2_HPO4 |  | 13 |  | 13 |  |
| CaCO_3_ |  | 5.5 |  | 5.5 |  |
| C_6_H_5_K_3_O_7_.1H_2_O |  | 16.5 |  | 16.5 |  |
| Vitamin mix V10001 |  | 10 |  | 10 |  |
| Choline Bitartrate |  | 2 |  | 2 |  |
| Energy (kcal/g) |  | 3.8 |  | 3.8 |  |
| Protein (% kcal) |  | 20 |  | 20 |  |
| Carbohydrate (% kcal of which sucrose) |  | 70 (35) |  | 70 (35) |  |
| Fat (% kcal) |  | 10 |  | 10 |  |

^a^ Diets formulated and produced by Research Diets Inc. (New Brunswick, NJ, USA)

CAS, casein; WPI, whey protein isolate.
