# Supplementary material for: The Sex Dependent and Independent Effects of Dietary Whey Proteins Are Passed from the Mother to the Offspring
Source: Mol Nutr Food Res. 2024 Nov 3;68(23):2400584. doi: 10.1002/mnfr.202400584 (PMC11653169; doi:10.1002/mnfr.202400584)
Supplement: Supplementary file 2 — Supporting information [file MNFR-68-2400584-s001.docx]

**Supplementary Table S2:** **The relative abundance of metabolites in the duodenum and colon in virgin mice fed casein (CAS) or whey protein isolate (WPI).**

|  | **Duodenum** | | | **Colon** | | |
| --- | --- | --- | --- | --- | --- | --- |
| **Nutrient** | **Virgin-CAS** | **Virgin-WPI** | **Significance**  **P<0.05 (FDR)** | **Virgin-CAS** | **Virgin-WPI** | **Significance P<0.05(FDR)** |
| Alanine | 478.6±56.7 | 530.5±45.5 | 0.49 | 236.2±30.5 | 222.0±26.9 | 0.73 |
| Glycine | 255±28.4 | 290.3±36 | 0.45 | 183.6±15.1 | 163.3±13.7 | 0.34 |
| Valine | 137.8±18.3 | 173±20.7 | 0.27 | 47.4±2.4 | 44.2±2.2 | 0.35 |
| Leucine | 161.4±16.1 | 233.8±24.8 | 0.05(1.2) | 37.8±2.5 | 35.1±1.9 | 0.4 |
| Threonine | 77.6±7.8 | 96.2±9.7 | 0.16 | 42.1±3.2 | 39.3±3.9 | 0.63 |
| Serine | 132.9±4±12.6 | 155.8±16.8 | 0.30 | 57.0±4.4 | 47.1±4.8 | 0.16 |
| Glutamic acid | 237±24.2 | 208.2±29.4 | 0.46 | 243.2±19.8 | 227.9±26.6 | 0.65 |
| Methionine | 59.3±5.3 | 74.2±8.0 | 0.15 | 16.8±2.0 | 13.5±1.5 | 0.23 |
| Phenylalanine | 138.1±13.1 | 186.9±17.5 | 0.07(1.44) | 26.3±1.7 | 23.0±2.1 | 0.25 |
| Ornithine | 18.8±2.1 | 22.9±2.5 | 0.25 | 3.8±0.4 | 3.5±0.4 | 0.53 |
| Lysine | 206.8±22 | 293±43.7 | 0.11 | 48.8±1.9 | 42.9±4.2 | 0.24 |
| Tryptophan | 20.1±2.7 | 27.6±4.4 | 0.18 | 4.9±0.6 | 3.9±0.5 | 0.25 |
| Asparagine | 45.0±4.7 | 51.5±6.0 | 0.42 | 117.3±11.7 | 96.3±15.3 | 0.30 |
| Tyrosine | 99.4±11.1 | 145.1±18.5 | 0.10 | 21.0±1.5 | 15.7±`1.4 | 0.031(1.46) |
| Acetic (C2:0) | 8.6±1.2 | 5.9±1.9 | 0.04(1.44) | 7.0±0.5 | 6.5±1.2 | 0.81 |
| Propionic (C3:0) | 0.22±0.03 | 0.14±0.05 | 0.01(0.72) | 0.6±0.05 | 0.33±0.03 | 0.002(0.28) |
| Butyric (C4:0n) | 0.36±0.09 | 0.18±0.04 | 0.07(1.44) | 0.61±0.09 | 0.48±0.15 | 0.53 |
| Valeric (C5:0n) | 0.10±0.01 | 0.08±0.01 | 0.22 | 0.51±0.04 | 0.40±0.09 | 0.42 |
| PC aa C34:2 | 114.8±12.6 | 166.0±13.3 | 0.02(0.96) | 58.0±7.0 | 59.5±5.1 | 0.86 |
| PC aa C38:1 | 0.51±0.14 | 0.6±0.09 | 0.63 | 1.01±0.10 | 0.76±0.06 | 0.05(1.77) |
| PC ae C34:0 | 0.92±0.05 | 0.75±0.14 | 0.12 | 1.52±0.11 | 1.13±0.08 | 0.02(1.42) |
| PC ae C42:3 | 1.4±0.15 | 0.88±0.06 | 0.005(0.72) | 0.63±0.04 | 0.53±0.03 | 0.08(2.27) |
| PC ae C44:5 | 0.52±0.03 | 0.39±0.04 | 0.046(1.32) | 0.28±0.02 | 0.23±0.03 | 0.28 |

The metabolomics data (mean ±SE) are shown for virgin female mice fed casein (CAS; n=8) or whey protein isolate (WPI; n=10) for 11 weeks. Data related to animals in each group and for each tissue, were analysed as independent biological replicates by unpaired t-test. Only selected metabolites are shown from the analysis that included 21 amino acids, 21 biogenic amines as well as 40 acylcarnitines, 14 lysophosphatidylcholines (lysoPCs), 76 phosphatidylcholines (PCs), 15 sphingomyelins (SMs), and the sum of hexoses (H1). False discover rate (FDR) is shown where P<0.05. Phosphatidylcholine diacyl (PC aa), Phosphatidylcholine acyl-alkyl (PC ae).
