# Supplementary material for: The Sex Dependent and Independent Effects of Dietary Whey Proteins Are Passed from the Mother to the Offspring
Source: Mol Nutr Food Res. 2024 Nov 3;68(23):2400584. doi: 10.1002/mnfr.202400584 (PMC11653169; doi:10.1002/mnfr.202400584)
Supplement: Supplementary file 3 — Supporting information [file MNFR-68-2400584-s002.docx]

**Supplementary Table S3:** **The relative abundance of caecal amino acids in virgin and lactating mice fed casein (CAS) or whey protein isolate (WPI)**

| **Nutrient** | **Virgin**  **CAS** | **Virgin**  **WPI** | **Lactating CAS** | **Lactating WPI** | **Significance at P<0.05(FDR)** | | |
| --- | --- | --- | --- | --- | --- | --- | --- |
|  |  |  |  |  | **Prot** | **Reprod** | **Inter:** |
| Cysteine | 1.14±0.25 | 0.57±0.16 | 1.98±0.78 | 0.41±0.13 | 0.005  (0.07) | 0.34 | 0.16 |
| Alanine | 0.91±0.14 | 1.14±0.11 | 1.24±0.14 | 1.24±0.12 | 0.40 | 0.14 | 0.41 |
| Glycine | 0.89±0.14 | 1.03±0.12 | 1.13±0.10 | 0.97±0.08 | 0.93 | 0.48 | 0.25 |
| Valine | 0.95±0.14 | 1.11±0.13 | 1.14±0.12 | 0.98±0.11 | 0.97 | 0.82 | 0.26 |
| Leucine | 0.98±0.14 | 1.13±0.13 | 1.19±0.21 | 0.98±0.13 | 0.83 | 0.82 | 0.25) |
| Threonine | 0.95±0.16 | 1.03±0.13 | 1.14±0.19 | 0.79±0.22 | 0.48 | 0.90 | 0.25 |
| Serine | 1.00±0.17 | 1.12±0.12 | 1.28±0.23 | 0.96±0.11 | 0.54 | 0.69 | 0.19 |
| Glutamic acid | 0.93±0.14 | 1.24±0.17 | 1.27±0.18 | 1.08±0.04 | 0.70 | 0.57 | 0.12 |
| Methionine | 0.99±0.14 | 1.14±0.12 | 1.15±0.17 | 1.00±0.12 | 0.99 | 0.95 | 0.30 |
| Phenylalanine | 0.97±0.14 | 1.13±0.12 | 1.15±0.17 | 1.03±0.13 | 0.88 | 0.74 | 0.33 |
| Ornithine | 0.77±0.18 | 0.95±0.15 | 1.71±0.47 | 1.49±0.46 | 0.95 | 0.034  (0.11) | 0.54 |
| Lysine | 0.94±0.15 | 1.17±0.11 | 1.27±0.18 | 0.95±0.20 | 0.79 | 0.74 | 0.12 |
| Tryptophan | 1.11±0.15 | 1.03±0.14 | 1.14±0.25 | 0.91±0.23 | 0.42 | 0.81 | 0.68 |
| Asparagine | 1.04±0.13 | 1.06±0.10 | 1.14±0.15 | 0.49±0.22 | 0.057 | 0.14 | 0.045(1.89) |
| Tyrosine | 0.96±0.14 | 1.13±0.13 | 1.15±0.17 | 1.02±0.14 | 0.89 | 0.78 | 0.31 |

The metabolomics data (mean±SE) are shown for virgin females fed CAS (n=8) or WPI (n=10) and for lactating females fed CAS (n=4) or WPI (n=6) for 11 weeks. Data related to animals in each group, were analysed as independent biological replicates by Univariate factorial ANOVA with post hoc analysis undertaken with Bonferroni. The significance is shown with regard to the effect of protein quality (Pro:), reproduction (Repro:) and their interactions (inter;). Only amino acids are shown from the analysis that included medium and long chain fatty acids. False discover rate (FDR) is shown where P<0.05.
