# Supplementary material for: The Sex Dependent and Independent Effects of Dietary Whey Proteins Are Passed from the Mother to the Offspring
Source: Mol Nutr Food Res. 2024 Nov 3;68(23):2400584. doi: 10.1002/mnfr.202400584 (PMC11653169; doi:10.1002/mnfr.202400584)
Supplement: Supplementary file 4 — Supporting information [file MNFR-68-2400584-s003.docx]

**Supplementary Table S4:** **The relative abundance of metabolites in the plasma of male and female offspring of mothers fed casein (CAS) or whey protein isolate (WPI)**

| **Nutrient** | **Male off**  **Moth.-CAS** | **Male off**  **Moth.-WPI** | **Female off Moth.-CAS** | **Female off**  **Moth.-WPI** | **Significance at P<0.05(FDR)** | | |
| --- | --- | --- | --- | --- | --- | --- | --- |
|  |  |  |  |  | **Prot:** | **Sex:** | **Inter** |
| Alanine | 3.24±0.09 | 2.97±0.18 | 2.73±0.21 | 2.81±0.22 | 0.61 | 0.08 | 0.37 |
| Glycine | 3.21±0.17 | 3.36±0.13 | 2.70±0.19 | 3.03±0.12 | 0.14 | 0.014(0.26) | 0.573 |
| Valine | 2.81±0.13 ^a^ | 2.29±0.17 ^b^ | 2.7±0.19 | 2.32±0.1 | 0.007(0.29) | 0.81 | 0.668 |
| Leucine | 2.58±0.19 | 2.01±0.21 | 2.58±0.35 | 1.97±0.17 | 0.021(0.44) | 0.943 | 0.923 |
| Threonine | 2.93±0.08 | 2.81±0.16 | 2.54±0.09 | 2.65±0.09 | 0.94 | 0.027(0.28) | 0.335 |
| Serine | 3.07±0.24 | 3.29±0.23 | 2.91±0.27 | 3.06±0.22 | 0.45 | 0.43 | 0.91 |
| Glutamic acid | 1.88±0.10 | 1.96±0.14 | 1.88±0.17 | 1.92±0.13 | 0.66 | 0.89 | 0.90 |
| Methionine | 3.04±0.08 | 2.78±0.13 | 2.70±0.07 | 2.61±0.14 | 0.137 | 0.042(0.35) | 0.49 |
| Phenylalanine | 2.93±0.10 | 2.67±0.13 | 2.70±0.09 | 2.67±0.08 | 0.19 | 0.29 | 0.29 |
| Ornithine | 2.84±0.45 | 3.12±0.43 | 2.48±0.55 | 2.55±0.32 | 0.69 | 0.30 | 0.81 |
| Lysine | 2.44±0.085 | 2.34±0.09 | 2.47±0.09 | 2.28±0.11 | 0.15) | 0.90 | 0.67 |
| Tryptophan | 1.43±0.13 | 1.27±0.11 | 1.42±0.19 | 1.42±0.11 | 0.57 | 0.62 | 0.59 |
| Asparagine | 2.5±0.21 | 2.37±0.23 | 2.380±0.23 | 2.24±0.14 | 0.41 | 0.44( | 0.85 |
| Tyrosine | 3.36±0.37 | 3.33±0.29 | 2.91±0.30 | 3.12±0.33 | 0.79) | 0.32 | 0.71 |
| Aspartic acid | 2.31±0.16 | 2.75±0.08 | 3.13±0.30 | 3.24±0.27 | 0.22 | 0.006(0.25) | 0.443 |
| Succinic | 2.45±0.23 | 2.24±0.25 | 2.55±0.92 | 3.11±0.94 | 0.79 | 0.47 | 0.56 |
| Lactic | 2.9±0.20 | 2.37±0.24 | 2.8±0.17 | 2.86±0.17 | 0.26 | 0.33 | 0.16 |
| Isocitric | 1.91±0.12 | 1.58±0.09 | 1.91±0.17 | 1.94±0.14 | 0.28 | 0.18 | 0.19 |
| Dodecanoic acid (C12:0) | 2.06±0.37 | 1.73±0.34 | 1.73±0.21 | 1.77±0.14 | 0.62 | 0.61 | 0.53 |
| Tetradecanoic acid (C13:0) | 2.89±0.43 | 3.014±0.63 | 3.06±0.43 | 3.04±0.32 | 0.92 | 0.83 | 0.87 |
| Palmitoleic acid (C16:1) | 3.22±0.32 | 3.29±0.48 | 3.2±0.38 | 3.39±0.36 | 0.74 | 0.91 | 0.88 |
| Hexadecanoic acid (C16:0) | 14.16±1.35 | 13.3±0.86 | 14.2±0.64 | 13.16±0.52 | 0.30 | 0.97 | 0.89 |
| Heptadecanoic acid | 3.08±0.28 | 2.69±0.14 | 2.86±0.09 | 2.56±0.10 | 0.073 | 0.43 | 0.95 |
| Stearic (C18:0) | 2.25±0.17 | 2.34±0.144 | 2.83±0.18 | 2.59±0.14 | 0.65 | 0.019(0.26) | 0.329 |
| Oleic acid (C18:1) | 2.55±0.20 | 2.44±0.15 | 2.69±0.1 | 2.56±0.09 | 0.42 | 0.38 | 0.97 |
| Linolenic acid (C18:3) | 3.45±0.25 | 3.19±0.38 | 3.4±0.31 | 3.52±0.33 | 0.82 | 0.66 | 0.56 |

The data (mean±SE) are shown for male offspring (off) (n=7) of mothers (moth) fed CAS, female offspring (n=10) of mothers fed CAS, male offspring (n=12) of mothers fed WPI and female offspring (n=13) of mothers fed WPI. Data related to animal in each group were analysed as independent biological replicates by Univariate factorial ANOVA with post hoc analysis undertaken with Bonferroni. The analysis was performed following feeding casein (CAS) enriched diet to all offspring for 4 weeks. The significance of data is shown with regard to the effect of protein quality (Pro:) of the mother’s diet, sex of the offspring and their interactions (inter;). Only selected fatty acids are shown. False discover rate (FDR) is shown where P<0.05.
