# Supplementary material for: The Sex Dependent and Independent Effects of Dietary Whey Proteins Are Passed from the Mother to the Offspring
Source: Mol Nutr Food Res. 2024 Nov 3;68(23):2400584. doi: 10.1002/mnfr.202400584 (PMC11653169; doi:10.1002/mnfr.202400584)
Supplement: Supplementary file 5 — Supporting information [file MNFR-68-2400584-s006.docx]

**Suppmentary Table S5: Significance of the correlation between metabolic parameters in male and female offspring of mothers fed casein (CAS) or whey protein isolate (WPI).**


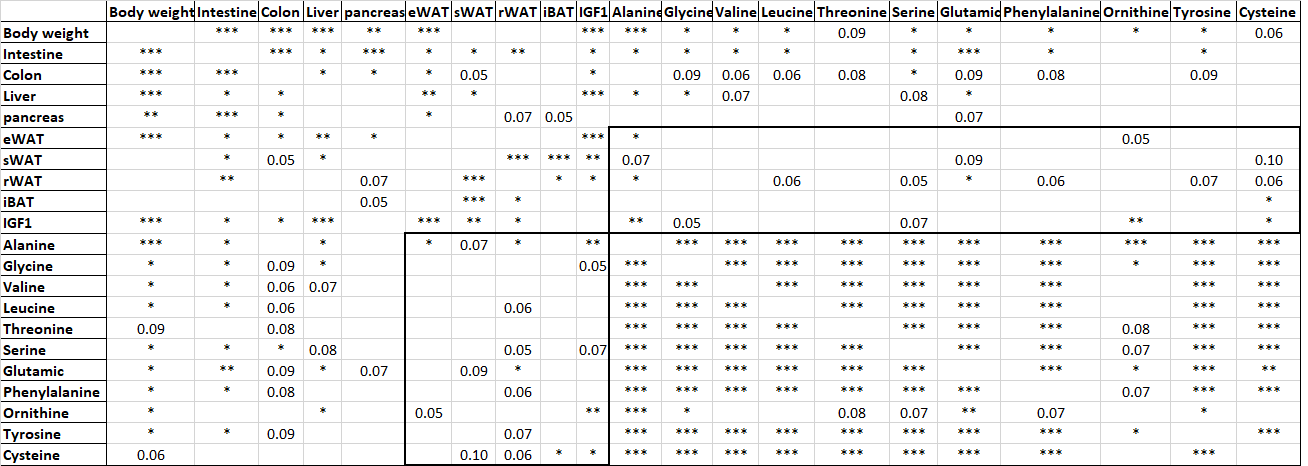


P values are shown for Pearson correlation between metabolic parameters of male offspring (n=7) of mothers fed CAS, female offspring (n=10) of mothers fed CAS, male offspring (n=12) of mothers fed WPI and female offspring (n=13) of mothers fed WPI. The analysis was performed following feeding casein enriched diet to all offspring for 4 weeks. Insulin-like growth factor-1 (IGF-1), gonadal white adipose tissue (gWAT), subcutaneous WAT (sWAT), retroperitoneal WAT (rWAT) and interscapular brown adipose tissue (iBAT). *P<0.05; **P<0.005; ***P<0.001.
