# Supplementary material for: The Sex Dependent and Independent Effects of Dietary Whey Proteins Are Passed from the Mother to the Offspring
Source: Mol Nutr Food Res. 2024 Nov 3;68(23):2400584. doi: 10.1002/mnfr.202400584 (PMC11653169; doi:10.1002/mnfr.202400584)
Supplement: Supplementary file 6 — Supporting information [file MNFR-68-2400584-s005.docx]

**Supplementary table S6:** **The impact of whey protein isolate relative to casein on the hypothalamic transcriptome in male mice.**

| **Gene ID** | **Gene symbol** | **Gene name** | **P adjusted** | **Fold change** |
| --- | --- | --- | --- | --- |
| ENSMUSG00000023083 | H2-M10.1 (includes others) | histocompatibility 2, M region locus 10.1 | 0.0271 | 2.489 |
| ENSMUSG00000089736 | TGFBR3L | transforming growth factor beta receptor 3 like | 0.0389 | 1.58 |
| ENSMUSG00000055632 | HMCN2 | hemicentin 2 | 0.00873 | 1.301 |
| ENSMUSG00000058740 | KCNT1 | potassium sodium-activated channel subfamily T member 1 | 0.0493 | 1.161 |
| ENSMUSG00000047036 | ZNF445 | zinc finger protein 445 | 0.0493 | 1.131 |
| ENSMUSG00000039218 | Srrm2 | serine/arginine repetitive matrix 2 | 0.0253 | 1.127 |
| ENSMUSG00000006154 | EPS8L1 | EPS8 like 1 | 0.0251 | 1.501 |
| ENSMUSG00000029622 | ARPC1B | actin related protein 2/3 complex subunit 1B | 0.0133 | -1.237 |
| ENSMUSG00000004665 | CNN2 | calponin 2 | 0.0342 | -1.304 |
| ENSMUSG00000030707 | CORO1A | coronin 1A | 0.00263 | -1.214 |
| ENSMUSG00000027368 | DUSP2 | dual specificity phosphatase 2 | 0.000029 | -2.876 |
| ENSMUSG00000030830 | ITGAL | integrin subunit alpha L | 0.00000969 | -5.35 |
| ENSMUSG00000001281 | ITGB7 | integrin subunit beta 7 | 7.84E-13 | -4.633 |
| ENSMUSG00000052837 | JUNB | JunB proto-oncogene, AP-1 transcription factor subunit | 0.0493 | -1.523 |
| ENSMUSG00000020437 | MYO1G | myosin IG | 0.0497 | -2.097 |
| ENSMUSG00000040435 | PPP1R15A | protein phosphatase 1 regulatory subunit 15A | 0.0009 | -1.392 |
| ENSMUSG00000020372 | RACK1 | receptor for activated C kinase 1 | 0.0000819 | -1.2 |
| ENSMUSG00000003970 | RPL8 | ribosomal protein L8 | 0.00948 | -1.11 |
| ENSMUSG00000059291 | RPL11 | ribosomal protein L11 | 0.0493 | -1.126 |
| ENSMUSG00000038900 | RPL12 | ribosomal protein L12 | 0.00143 | -1.137 |
| ENSMUSG00000000740 | RPL13 | ribosomal protein L13 | 0.000288 | -1.137 |
| ENSMUSG00000059070 | RPL18 | ribosomal protein L18 | 0.00191 | -1.164 |
| ENSMUSG00000073702 | RPL31 | ribosomal protein L31 | 0.031 | -1.126 |
| ENSMUSG00000045128 | RPL18A | ribosomal protein L18a | 0.00561 | -1.149 |
| ENSMUSG00000062006 | Rpl34 (includes others) | ribosomal protein L34 | 0.0317 | -1.134 |
| ENSMUSG00000046330 | RPL37A | ribosomal protein L37a | 0.0455 | -1.132 |
| ENSMUSG00000067274 | RPLP0 | ribosomal protein lateral stalk subunit P0 | 0.00242 | -1.183 |
| ENSMUSG00000025508 | RPLP2 | ribosomal protein lateral stalk subunit P2 | 0.0378 | -1.131 |
| ENSMUSG00000030744 | RPS3 | ribosomal protein S3 | 0.0094 | -1.134 |
| ENSMUSG00000012848 | RPS5 | ribosomal protein S5 | 0.0418 | -1.118 |
| ENSMUSG00000061983 | RPS12 | ribosomal protein S12 | 0.0389 | -1.111 |
| ENSMUSG00000024608 | RPS14 | ribosomal protein S14 | 0.0114 | -1.152 |
| ENSMUSG00000037563 | RPS16 | ribosomal protein S16 | 0.00109 | -1.154 |
| ENSMUSG00000008668 | RPS18 | ribosomal protein S18 | 0.0378 | -1.169 |
| ENSMUSG00000032518 | RPSA | ribosomal protein SA | 0.00561 | -1.103 |
| ENSMUSG00000004815 | DGKQ | diacylglycerol kinase theta | 0.00285 | 1.159 |
| ENSMUSG00000034171 | FAAH | fatty acid amide hydrolase | 0.0349 | 1.169 |
| ENSMUSG00000016252 | Atp5e | ATP synthase, H+ transporting, mitochondrial F1 complex, epsilon subunit | 0.031 | -1.172 |
| ENSMUSG00000032259 | DRD2 | dopamine receptor D2 | 0.0389 | -1.286 |
| ENSMUSG00000064357 | MT-ATP6 | ATP synthase F0 subunit 6 | 0.0014 | -1.278 |
| ENSMUSG00000064354 | MT-CO2 | cytochrome c oxidase subunit II | 0.00249 | -1.208 |
| ENSMUSG00000001348 | Acp5 | acid phosphatase 5, tartrate resistant | 0.00311 | -3.518 |
| ENSMUSG00000060802 | B2M | beta-2-microglobulin | 0.0493 | -1.19 |
| ENSMUSG00000035042 | CCL5 | C-C motif chemokine ligand 5 | 0.0378 | -4.938 |
| ENSMUSG00000000682 | Cd52 | CD52 antigen | 0.0389 | -2.545 |
| ENSMUSG00000090877 | Hspa1b | heat shock protein 1B | 2.61E-08 | -1.816 |
| ENSMUSG00000030742 | LAT | linker for activation of T cells | 0.031 | -2.959 |
| ENSMUSG00000024399 | LTB | lymphotoxin beta | 7.84E-13 | -4.72 |
| ENSMUSG00000033220 | RAC2 | Rac family small GTPase 2 | 3.2E-10 | -3.293 |
| ENSMUSG00000044199 | S1PR4 | sphingosine-1-phosphate receptor 4 | 0.0142 | -2.609 |
| ENSMUSG00000000486 | SEPTIN1 | septin 1 | 0.00000324 | -2.529 |
| ENSMUSG00000019872 | SMPDL3A | sphingomyelin phosphodiesterase acid like 3A | 0.00948 | -1.247 |
| ENSMUSG00000033450 | TAGAP | T cell activation RhoGTPase activating protein | 0.0133 | -2.373 |
| ENSMUSG00000000782 | Tcf7 | transcription factor 7, T cell specific | 0.000116 | -1.727 |
| ENSMUSG00000019850 | TNFAIP3 | TNF alpha induced protein 3 | 1.53E-08 | -2.434 |
| ENSMUSG00000060550 | HLA-A | major histocompatibility complex, class I, A | 6.98E-08 | -2.503 |
| ENSMUSG00000001588 | ACAP1 | ArfGAP with coiled-coil, ankyrin repeat and PH domains 1 | 0.0457 | -2.32 |
| ENSMUSG00000040345 | ARHGAP9 | Rho GTPase activating protein 9 | 0.000227 | -2.478 |
| ENSMUSG00000000409 | LCK | LCK proto-oncogene, Src family tyrosine kinase | 0.00000324 | -2.51 |
| ENSMUSG00000040128 | PNRC1 | proline rich nuclear receptor coactivator 1 | 0.00873 | -1.146 |
| ENSMUSG00000025869 | NOP16 | NOP16 nucleolar protein | 0.0402 | -1.193 |
| ENSMUSG00000025647 | SHISA5 | shisa family member 5 | 0.0191 | -1.163 |
| ENSMUSG00000006442 | SRM | spermidine synthase | 0.0493 | -1.153 |
| ENSMUSG00000060126 | TPT1 | tumor protein, translationally-controlled 1 | 0.00232 | -1.128 |
| ENSMUSG00000044786 | ZFP36 | ZFP36 ring finger protein | 0.000562 | -1.827 |
| ENSMUSG00000045817 | ZFP36L2 | ZFP36 ring finger protein like 2 | 0.0415 | -1.165 |

The data were generated following application of RNAseq on archived tissues from a previously published study involving male mice fed whey proteins or casein (n=8) [8]. Positive numbers refer to increased and negative numbers refer to decreased expression. P values were adjusted for multiple comparisons using a Benjamini and Hochberg (B-H) method.
